# The Iodine Content in Urine, Faeces and Selected Organs of Rats Fed Lettuce Biofortified with Iodine Through Foliar Application

**DOI:** 10.1007/s12011-016-0717-0

**Published:** 2016-04-30

**Authors:** Roksana Rakoczy, Aneta Kopeć, Ewa Piątkowska, Sylwester Smoleń, Łukasz Skoczylas, Teresa Leszczyńska, Włodzimierz Sady

**Affiliations:** 1 Unit of Plant Nutrition, Institute of Plant Biology and Biotechnology, Faculty of Biotechnology and Horticulture, University of Agriculture in Krakow, Al. 29 Listopada 54, 31-425, Krakow, Poland; 2 Department of Human Nutrition, Faculty of Food Technology, University of Agriculture in Krakow, Balicka 122, 30-149, Krakow, Poland; 3 Department of Fruit, Vegetable and Mushroom Processing, Faculty of Food Technology, University of Agriculture in Krakow, Balicka 122, 30-149, Krakow, Poland

**Keywords:** Biofortification, Foliar application, Iodine, Lettuce, Rats

## Abstract

Iodine is an essential trace element for humans. Foliar application of micronutrients is successfully used in order to increase the concentration of essential elements in vegetables. The aim of this study was to evaluate the iodine absorption in the rat organism fed foliar biofortified lettuce. The presented study was consisted of the vegetative and animal experiment. In the vegetative experiment with lettuce, two combinations of foliar application were used: (1) control—without iodine application and (2) iodine application in the potassium iodide (KI) form. In the animal experiment, Wistar rats were divided to four groups, which received one of four diets: (1) C—control diet containing iodine in the KI form, (2) D—diet deficient in iodine, (3) D + BL—diet containing biofortified lettuce, and (4) D + CL—diet containing control lettuce (as the only source of iodine in diet, respectively). The diets contained 0.260, 0.060, 0.254 and 0.075 mg I/kg, respectively. In order to determine the iodine absorption in the rat organisms, the content of this trace element was measured in urine, faeces and in selected organs with the use of the ICP-OES technique. Foliar application of the KI increased the content of iodine in lettuce. The rats from the D + BL group excreted significantly less iodine in their urine and faeces and also accumulated more iodine in the organs than the rats from the C group. Iodine with biofortified lettuce was much bioavailable for rodents than iodine from control diet. Biofortified lettuce can be a source of iodine in a diet of human and can improve iodine nutrition.

## Introduction

Iodine is a very important micronutrient for the proper functioning of thyroid hormones, which regulate the metabolism and has crucial impact on brain development in humans and animals. Iodine deficiency (ID) causes health problems known as iodine deficiency disorders (IDD) such as goitre, hypothyroidism, foetus anomalies and mental retardation [1–3]. Further, even moderate ID results in loss of 10–15 IQ points at a population level that indirectly affects the development of whole nations [1].

The World Health Organization (WHO) and United Nations International Children’s Emergency Fund (UNICEF) have recommended the commonly implemented Universal Salt Iodization (USI) programme to counteract consequences of IDD. This programme has been a safe, efficient and cost-effective method of elimination of iodine deficiency in many countries [4, 5]. The progress in reducing the ID problem in the world was made in 2003–2011. In this period, the number of iodine-deficient countries decreased from 54 to 32 and the number of countries with adequate iodine intake increased from 67 to 105. Currently, 71 % of the population in the world has access to iodized salt, up from 20 % in 1990. However, despite this remarkable progress, around 1.9 billion people including 241 million school-age children are still at risk of ID [5, 6].

In less-industrialised countries, where salt iodization programmes have been introduced, progress in reduction of ID is observed [7]. However, in some industrialised countries, iodine intake has fallen and problem of ID has reappeared. The governments should conduct systematic assessment and monitoring of ID in populations, and every country should have a prophylaxis programme to address ID. Presently, half of the European population is mildly ID as a result of weak or non-existent iodine prophylaxis in Europe. Approximately 90 % of the salt consumption in industrialised countries is from processed food, in which salt is not always iodized [8]. Moreover, epidemiological research has shown that high salt intake is associated with significantly increased risk of stroke and other cardiovascular diseases [9]. The level of salt consumption exceeds the amount recommended by the WHO (5 g NaCl/day). In Europe and Asia, daily average consumption of salt is higher than 12 g [9, 10]. In the years 2008–2013, the WHO implemented the programme ‘Global Strategy on Diet, Physical Activity and Heath’, aimed at the reduction of salt consumption and identification of alternative ways of iodine introduction into food [11]. One of these methods can be biofortification of vegetables with iodine, through foliar application during cultivation [12, 13]. In human diet, over 80 % of iodine is from vegetal foodstuffs. Almost 100 % of iodine in food is bioavailable to humans [14]. Therefore, biofortification of vegetables with iodine could be an effective way to reduce and control ID in developed and developing countries. Lettuce was chosen as a good candidate for biofortification study because of the leaves—edible parts of this vegetable store a lot of iodine. Lettuce is almost always eaten raw with no risk of iodine loss during cooking. In addition, lettuce can be cultivated year-round.

The objective of this study was to evaluate the effect of the addition of the foliar biofortified lettuce with iodine in the potassium iodide (KI) form into rats’ diet and the iodine content in urine, faeces, selected organs as well as the thyroid-stimulating hormone (TSH) concentration in Wistar rats.

## Materials and Methods

### Plant Material

The experiment with hydroponic cultivation of lettuce cv. ‘Melodion’ was conducted in the spring season of 2012 in non-heated foil tunnel belonging to the Faculty of Biotechnology and Horticulture, University of Agriculture. The seedlings were produced in a greenhouse. Lettuce seeds were sown into rockwool plugs in the first half of March (12 March 2012). The seedlings were placed on rockwool blocks (10 × 10 × 6.5 cm) in a two-leaf phase. After rooting, plants in rockwool blocks were transferred to the foil tunnel. The plants received a nutrient solution with the following content of macro- and micronutrients (mg/dm^3^): 120 N, 25 P, 160 K, 40 Mg, 140 Ca, 0.8 Fe, 0.6 Mn, 0.3 Zn, 0.2 B, 0.1 Cu, 0.02 Mo, pH 6.0 and EC 1.6 mS/cm. The nutrient solution was applied by drip irrigation system in an open cycle (without recirculation of nutrient solution).

The lettuce plants were divided randomly into two combinations, the first with foliar application of distilled water (control) and the second with foliar application of iodine in the KI form which was soluted in distilled water. Both combinations were consisted of four replicates with 14 plants in each replicate. Foliar application was conducted thrice, every 7 days from the phase of six leaves till 7 days before the harvest. The concentration of iodine used for spraying was 0.001 % (10 mg/dm^3^). Totally, for three treatments, 107 cm^3^ of water or KI solution was used to each lettuce plant. Each head of lettuce received 1.07 mg iodine applied foliarly. At harvest (16 May 2012), representative samples of lettuce plants from both combinations were collected for the animal experiment.

### Analysis in Plant Material

Fresh samples of lettuce were frozen and freeze-dried with lyophilizer (Christ Alpha 1–4, Gefriertrocknungsanlangen, Germany). In such prepared samples, the chemical composition was estimated. Total proteins (method no. 950.36), raw fat (method no. 935.38), total dietary fibre (method no. 991.43) and ash (method no. 930.05) were measured according to the AOAC [15] methods.

The nitrates (V) (NO_3_^−^) content was analysed after extraction of 5 g fragmented fresh lettuce samples in 100 cm^3^ of 2 % acetic acid (aqueous solution of acetic acid). The NO_3_^−^ concentration was measured by the Flow Injection Analysis technique (FIA technique, FIA system, MLE GmbH Dresden, Germany) [16].

In order to analyse iodine content, air-dried lettuce samples were ground in a variable speed rotor mill Pulverisette 14 FRITSCH (Idar-Oberstein, Alemania, Germany) using a 0.5-mm sieve. Digestion of 0.5 g samples of lettuce in the mixture of 10 cm^3^ 65 % HNO_3_ and 0.8 cm^3^ 70 % HClO_4_ was conducted in the microwave system MARS-5 Xpress (CEM, World Headquarters, Matthews, NC, USA). The content of iodine was analysed by the cold vapour generation technique with the use of high-dispersion inductively coupled plasma optical emission spectrometry (ICP-OES, Prodigy spectrometer, Leeman Labs, USA) [17, 18]. A similar method was used for the determination of iodine content in the experimental diets of rats. To evaluate the accuracy of the analysis, the content of iodine in certified spinach leaves reference material (NCS ZC73013) was additionally determined. The obtained result was 0.39 ± 0.08 mg I/kg d.m., while the certified value was 0.36 ± 0.12 mg I/kg d.m.

### Animal Study

Four-week-old male Wistar rats (*n* = 32), with an average body mass of 123 ± 10 g, were purchased from Animal Husbandry in Brwinów, Warsaw, Poland. Experimental procedures were approved by the First Local Ethical Committee on Animal Testing at the Jagiellonian University in Krakow (Poland, res. no 57/2012). The rats were acclimatised for 1 week on standard laboratory chow, before the experiment. After that, the rats were randomly divided into four experimental groups (*n* = 8).

Experimental diets have been prepared on the basis of the AIN-93G diet [19]. Detailed description of diets is reported in Table 1. In the first group, the rats were fed control diet (C) in which the mineral mixture contained the iodine (in the KI form) in an amount recommended by Reeves [19]. In the second group, diet deficient in iodine (D) was prepared with mineral mixture without iodine. In the next group, diet deficient in iodine contained biofortified lettuce (D + BL)—lettuce as the only source of iodine (mineral mixture without iodine) was used. Biofortified lettuce was added to the D + BL in order to obtain the same amount of iodine as in the C diet. Diet deficient in iodine of the fourth group of rats contained control (non-biofortified) lettuce (D + CL) (mineral mixture without iodine). The level of iodine in the mineral mixture of D diet was the same as in the diet with non-biofortified lettuce (D + CL) (Table 1).

**Table 1:** Ingredients of experimental diets

Ingredient (g/kg)	C	D	D + BL	D + CL
Corn starch	532.486	532.486	528.446	528.746
Saccharose	100	100	100	100
Casein	200	200	200	200
Soybean oil	70	70	70	70
Fibre	50	50	48.70^a^	48.40^b^
Vitamin mix	10	10	10	10
Mineral mix	35	35^c^	35^d^	35^d^
Choline chloride	2.50	2.50	2.50	2.50
TBHQ^e^	0.014	0.014	0.014	0.014
Biofortified lettuce^f^	−	−	5.34	−
Control lettuce^f^	−	−	−	5.34
Iodine (mg/kg)	0.260	0.060	0.254	0.075

*C* control diet (AIN-93G), *D* diet deficient in iodine, *D + BL* diet containing biofortified lettuce, *D + CL* diet containing control lettuce

^a^1.3 g of fibre was delivered from biofortified lettuce

^b^1.6 g of fibre was delivered from control lettuce

^c^In mineral mix the level of iodine was the same as in control lettuce

^d^Mineral mix without iodine—in these diets the source of iodine was biofortified or control lettuce

^e^ *tert*-butylhydroquinone

^f^Freeze-dried lettuce

The rats were housed separately in stainless steel metabolic cages at 21 °C and 12/12-h light/dark cycle. During the experiment, animals had free access to deionised distilled water. The intake of experimental diets was recorded every day. Body weight gain was recorded during the whole experiment on a weekly basis.

Urine and faeces were collected between the 8th–12th (5 days in the second week of experiment) and 22th–26th (5 days in the fourth week of the experiment) day of the experiment to assess iodine excretion. Collected urine and faeces were kept separately at −20 °C until the analyses. After 5 weeks of experimental period, fasted rats were sacrificed by decapitation, blood was obtained by heart puncture and collected in plain test tubes. Blood samples were collected to obtain serum by centrifugation (1500×*g*, 15 min.). Kidneys, livers, hearts and femoral muscles were dissected, washed in 0.9 % sodium chloride, dried with laboratory tissue paper and weighed. Serum and organ samples were kept frozen at −80 °C until the analysis.

### Analysis in Serum and Blood

The level of TSH was measured in the serum with a Rat TSH ELISA kit (cat. no RTC007R BioVendor, BioVendor Laboratorini medicina, Brno, Czech Republic). The serum was analysed for the concentration of total cholesterol (TC) (cat no. Liquick Cor-CHOL60 2–204, PZ Cormay S.A. Lublin, Poland), high-density lipoprotein (HDL) cholesterol (cat no. Cormay HDL 2–053, PZ Cormay S.A. Lublin, Poland) and triacylglycerols (TAG) (cat no. Liquick Cor-TG60 2–253, PZ Cormay S.A. Lublin, Poland). The differences between TC and HDL were used for calculations of the amount of low-density lipoprotein and very low-density lipoprotein (LDL + VLDL) cholesterol level [20]. The level of glucose was measured in the whole blood with a glucometer (Accu-chek, Roche Diagnostic, Mannheim, Germany). The activity of alanine aminotransferase (ALT) and aspartate aminotransferase (AST) in the serum was measured using Alpha Diagnostic kits (cat no. TR70121 and TR71121, respectively; Alpha Diagnostic, Warsaw, Poland). The concentration of malondialdehyde (MDA) in the serum was measured using colorimetric assay with thiobarbituric acid [21]. Standard curve was used to determine the concentration of MDA.

### Analysis of Crude Lipid and MDA Level in the Liver

The level of crude fat was determined by the Soxhlet method with a Soxtec Avanti’s 2050 Auto Extraction Unit (Tecator Foss, Hillerød, Sweden) according to Kopeć et al. [22]. The concentration of MDA in the liver homogenate was measured using colorimetric assay with thiobarbituric acid [21]. Standard curve was used to determine the concentration of MDA.

### Iodine Content in Urine, Faeces and Selected Organs

Collected samples of urine were adjusted to the same volume before analysis. The faeces and organs (kidneys, livers, hearts and femoral muscles) were freeze-dried. After freeze-drying, the organs were weighed and crushed in mortal and pestle. Thus prepared samples (particle size about 1 mm) were used for measurements of iodine content. The content of iodine in these samples was analysed by the cold vapour generation technique with the use of ICP-OES [17, 18] after sample digestion in the mixture of 10 cm^3^ 65 % HNO_3_ and 0.8 cm^3^ 70 % HClO_4_ in the microwave system CEM MARS-5 Xpress.

### Statistical Analysis

The data was presented as mean ± SD. ANOVA with the Tukey test was used for testing the differences between experimental treatments. For the iodine content in urine or faeces, a two-way MANOVA with the Tukey test was used to study the effects between treatment and week (second and fourth) of experiment, *P* ≤ 0.05 (Statistica v. 12.5 StatSoft, Inc., Tulsa, OK, USA).

## Results

The foliar application of iodine in the form of KI in the hydroponic cultivation of lettuce did not affect the basic chemical composition of lettuce plants, compared to the control plants. However, the content of NO_3_^−^ was significantly lower in biofortified lettuce in comparison to non-biofortified lettuce. The level of iodine was significantly higher in the biofortified lettuce than in the control plants (Table 2).

**Table 2:** Basic chemical composition of lettuce used for animal experiment

Ingredient		(g/100 g d.m.)	
		Control lettuce	Biofortified lettuce
Protein		29.08 ± 2.27 a	21.39 ± 4.90 a
Dietary fibre		32.67 ± 2.37 a	25.50 ± 0.08 a
Crude fat		3.10 ± 0.04 a	2.95 ± 0.48 a
Ash		12.63 ± 0.16 a	13.65 ± 0.41 a
Nitrates(V) (mg/kg f.m.)		1429.80 ± 32.15 b	1255.90 ± 24.36 a
Iodine	(mg/kg d.m.)	8.70 ± 0.12 a	35.79 ± 0.66 b
	(mg/kg f.m.)	0.49 ± 0.01 a	2.06 ± 0.03 b

Data are presented as mean ± standard deviation

Values in rows with different letters (a, b) are significantly different, *P* ≤ 0.05

The experimental diets did not affect body weight gain, food efficiency ratio (FER) and weight of kidneys, liver, heart and femoral muscle of rats (Table 3).

**Table 3:** Body gain, feed efficiency ratio, kidney, liver, heart and femoral muscle weights of experimental rats

Treatment	C	D	D + BL	D + CL
Body gain (g)	182 ± 13	176 ± 13	173 ± 16	181 ± 11
FER	0.42 ± 0.01	0.38 ± 0.02	0.40 ± 0.01	0.42 ± 0.00
Kidneys (g)^a^	2.31 ± 0.05	2.17 ± 0.06	2.29 ± 0.06	2.32 ± 0.07
Liver (g)	11.9 ± 0.50	10.8 ± 0.24	10.4 ± 0.25	10.9 ± 0.58
Heart (g)	0.99 ± 0.02	1.05 ± 0.02	0.97 ± 0.01	1.05 ± 0.02
Femoral muscle (g)	9.23 ± 0.60	9.79 ± 1.42	10.41 ± 0.84	10.57 ± 0.98

Data are presented as mean ± standard deviation

*C* control diet, *D* diet deficient in iodine, *D + BL* diet containing biofortified lettuce, *D + CL* diet containing control lettuce, *FER* feed efficiency ratio (g) body weight gain/diet consumed (g)

No significant differences were observed for all the parameters, *P* > 0.05

^a^Weight of both kidneys

On the other hand, the experimental diets had a significant effect on selected biochemical parameters in rodents (Table 4). The level of TSH in the serum of rats fed the C and D + BL diets was significantly lower, compared to rats fed the D and D + CL diets. The concentration of TC was the highest in the serum of rats fed diet with biofortified lettuce. The level of TC was higher in the rats of group D + BL, in comparison to the D group. The content of LDL + VLDL cholesterol in the serum of rodents group D + BL was significantly higher than in the other experimental groups. The HDL level in the serum of the rats was not affected by the various dietary treatments. The TAG concentration in the serum as well as the glucose in the blood of the rats fed diets with biofortified and non-biofortified lettuce was significantly lower than in those fed C and D diets. The highest level of TAG was measured in the serum of rats fed the C diet. The content of crude fat in the liver of D group rats was significantly higher, compared to the C and D + BL groups. Various dietary treatments did not affect the activity of ALT and AST in the serum of rats. Statistically significant differences in the concentration of MDA in the liver and in the serum of the rats fed various experimental diets were also not found (Table 4).

**Table 4:** Selected biochemical parameters in rats fed experimental diets

Treatment	C	D	D + BL	D + CL
TSH (ng/mL)	0.51 ± 0.00 a	0.86 ± 0.10 b	0.49 ± 0.00 a	0.82 ± 0.02 b
TC (mmol/L)	1.33 ± 0.07 ab	1.11 ± 0.10 a	1.55 ± 0.09 b	1.29 ± 0.11 ab
HDL (mmol/L)	1.19 ± 0.04 a	1.00 ± 0.08 a	1.05 ± 0.07 a	1.00 ± 0.07 a
LDL + VLDL (mmol/L)	0.14 ± 0.03 a	0.11 ± 0.02 a	0.49 ± 0.06 b	0.28 ± 0.05 a
TAG (mmol/L)	0.71 ± 0.01 c	0.54 ± 0.01 b	0.46 ± 0.01 a	0.41 ± 0.02 a
Crude fat (g/100 g f.m.)^a^	8.83 ± 0.38 a	13.04 ± 1.50 b	8.76 ± 0.21 a	11.12 ± 0.70 ab
Glucose (mg/dL)^b^	135 ± 2.25 b	134 ± 4.78 b	123 ± 1.20 a	120 ± 3.59 a
ALT (U/L)	27.06 ± 1.91 a	26.93 ± 1.33 a	20.45 ± 1.51 a	25.19 ± 1.36 a
AST (U/L)	67.09 ± 5.36 a	74.45 ± 6.55 a	70.58 ± 6.24 a	74.95 ± 2.48 a
MDA (nmol/mL)^c^	0.74 ± 0.04 a	0.88 ± 0.05 a	0.77 ± 0.77 a	0.76 ± 0.76 a
MDA (nmol/mL)^d^	0.45 ± 0.11 a	0.27 ± 0.05 a	0.48 ± 0.04 a	0.28 ± 0.06 a

Data are presented as mean ± standard deviation

*C* control diet, *D* diet deficient in iodine, *D + BL* diet containing biofortified lettuce, *D + CL* diet containing control lettuce

Values in rows with different letters (a, b) are significantly different, *P* ≤ 0.05

^a^In the liver

^b^In whole blood

^c^In the liver homogenate

^d^In the serum

The experimental diets had a significant effect on iodine excretion with urine and faeces in rats (Table 5). The highest iodine excretion was measured in urine and faeces of rats fed the C diet. The concentration of iodine in urine and faeces of groups fed C and D + BL diets was higher than groups fed D and D + CL diets. Significant differences in excretion of iodine with urine or faeces between the second and the fourth week were observed. The rats fed C diet excreted more iodine with urine in the second week in comparison to the fourth week, while rats of the third group excreted faeces with more iodine in the second week compared to the fourth week (Table 5).

**Table 5:** Iodine content in urine, faeces and selected organs in rats

Treatment	C	D	D + BL	D + CL
Urine (μg I/dm^3^)^*^				
Second week	240.36 ± 0.26 d	55.72 ± 0.34 a	188.54 ± 2.33 b	54.25 ± 0.40 a
Fourth week	230.52 ± 3.35 c	54.07 ± 0.16 a	186.26 ± 1.87 b	55.18 ± 0.18 a
Faeces (μg I/kg d.m.)^*^				
Second week	1714 ± 0.03 c	537 ± 0.03 a	1366 ± 0.00 c	522 ± 0.03 a
Fourth week	1691 ± 0.03 c	618 ± 0.00 a	1227 ± 0.00 b	585 ± 0.02 a
Organs (mg I/kg d.m.)^**^				
Kidney	18.87 ± 0.27 b	4.11 ± 0.10 a	22.37 ± 0.09 c	4.65 ± 0.07 a
Liver	4.28 ± 0.02 c	1.07 ± 0.02 a	5.41 ± 0.08 d	1.32 ± 0.03 b
Heart	4.60 ± 0.15 c	1.46 ± 0.04 a	6.14 ± 0.01 d	1.82 ± 0.01 b
Femoral muscle	1.19 ± 0.02 b	0.14 ± 0.02 a	2.31 ± 0.05 c	0.21 ± 0.04 a

Urine and faeces were collected (separately) for 5 days in the second and fourth week of experiment. Data are presented as mean ± standard deviation

*C* control diet, *D* diet deficient in iodine, *D + BL* diet containing biofortified lettuce, *D + CL* diet containing control lettuce

^*^Two-way MANOVA was used for testing the effects of treatment and week of experiment; values with different letters (a, b) are significantly different, *P* ≤ 0.05

^**^Values in rows with different letters (a, b) are significantly different, *P* ≤ 0.05

Significant differences in the concentration of iodine in selected organs were also found between the experimental groups of rats (Table 5). The rodents fed the diet with addition of biofortified lettuce (D + BL) accumulated the highest content of iodine in the kidneys, liver, heart and femoral muscle. The concentration of iodine in the organs of rats fed D and D + CL diets was lower, compared to C and D + BL diets. The rats from the D + CL group accumulated more iodine in the liver and heart than the rats from the D group (Table 5).

## Discussion

The main objective of biofortification is increasing the content of the mineral elements in edible tissues of crop plants. This action is focused on the nutrients commonly lacking in human diets, i.e.: Fe, Zn, Cu, Ca, Mg, Cu, I or Se [23, 24]. Foliar nutrition of plants described also as foliar feeding, fertilisation or application is one of the most efficient and the fastest methods of plants enrichment in these elements [25]. The agronomic methods of biofortification of leafy vegetables with iodine allow to achieve satisfying results of the iodine concentration in edible parts of these plants, making them good candidates for iodine source in the human diet. The previously published researches showed successful results of enrichment of spinach [26, 27] lettuce [28, 29] or pakchoi [30] with iodine. Weng et al. [14] reported that the leafy vegetables store more iodine in edible tissues than the fruit vegetables, which is even about 70-fold more. Because of the controllable and efficient systems for crop growing, the hydroponic cultivation makes a great possibility for biofortification with iodine [29, 31, 32]. This type of cultivation also allows for year-round production of vegetables. Lettuce is one of the most commonly grown hydroponic vegetables and is very effective in the uptake of iodine [29]. In this study, the foliar application of iodine in the form of iodide (I^−^) was an efficient method of lettuce biofortification grown in a hydroponic system. The level of iodine in biofortified lettuce was higher, about 420 %, compared to control plants. Simultaneously, foliar application of iodine did not affect the chemical composition of lettuce (beside the content of NO_3_^−^). In the Kopeć et al. [33] study, soil fortification of lettuce with iodine also increased the concentration of this trace element in plants and had no effect on chemical parameters of plants. Lettuce heads accumulates a lot of NO_3_^−^, which in the organism can interfere with iodine metabolism by inhibiting iodine uptake by the thyroid gland, which leads to the development of goitre in humans [34] and animals [35]. Interesting is the fact that in this study significant reduction of NO_3_^−^ content in lettuce leaves was affected by foliar application of the KI. Blasco et al. [36] also showed that lettuce biofortified with I^−^ accumulated less NO_3_^−^ in comparison to control plants. It can be suggested that biofortification of lettuce with I^−^ inhibits metabolism of NO_3_^−^ in plants.

Lettuce is eaten as a fresh salad. The average daily consumption of lettuce is around 50 g [29]. In our study, biofortified lettuce, which was produced in this experiment, contained 103 μg I in 50 g fresh mass of leaves. Therefore, it is 70 % of the recommended daily intake (RDI) of iodine, which is 150 μg for adult persons per day. Consequently, supplementation of diet in foliar biofortified lettuce may improve human nutrition in iodine. Moreover, greenhouse hydroponic production of plants enriched with iodine by foliar application makes them available for all seasons of the year. These plants may be an alternative to iodized salt and part of iodine prophylaxis.

The evaluation of bioavailability of iodine in the body of rats with biofortified lettuce can prove the effectiveness of lettuce foliar biofortification with iodine. The diets used in the experiment were properly balanced, and the rats consumed whole daily portion of the diet. The addition of biofortified and non-biofortified lettuce to the diets had no effect of FER, body gain and weights of the kidneys, liver, heart as well as femoral muscle of rats. In addition, Kopeć et al. [33] observed that the addition of biofortified lettuce (cultivated in filed condition) with iodine to diet of rats was safe for them.

The diagnosis of ID is based on the analysis of the thyroid goitre development, examination of the concentration of TSH, as well as the concentration of iodine in urine [2, 4]. Two methods of ID diagnosis were used in this study. The first was analysis of the level of thyroid-stimulating hormone which is the biochemical marker of thyroid gland function and its hormones. The TSH increases the uptake of iodine by the thyroid gland and stimulates secretion of the hormones thyroxine (T4) and triiodothyronine (T3) to the blood [37]. The increased TSH level can indicate the T4 and T3 synthesis disorder and on the insufficient supply of iodine in the diet. In the rats fed control diet (C) and diet with addition of biofortified lettuce (D + BL), the level of TSH was significantly lower than in the second and the fourth group of rats (D and D + CL). It indicates that iodine from biofortified lettuce was bioavailable for rats and was consequently used for the synthesis of thyroid hormones, whereas the increased TSH level in the serum of rats from the D and D + CL groups indicated an insufficient level of iodine in these diets, compared to the requirements of rats of this trace element.

The concentration of the TC and the LDL + VLDL cholesterol was the highest in the serum of rats fed diet containing biofortified lettuce (D + BL), while the level of accumulation of crude fat in the liver of this group was the lowest. Probably, it resulted from the fact that biologically active components in the lettuce, such as poliphenolics and short-chain fatty acids produced from lettuce fibre in the colon by bifidobacteria could inhibit the triacylglycerols and cholesterol accumulation in the liver [33]. However, the level of LDL + VLDL cholesterol in the serum of rats fed non-biofortified lettuce (D + CL) was not affected by addition of lettuce. In the rats, the high level of serum HDL and low LDL is connected with the fact that in the blood cholesterol is transported mainly in high-density lipoproteins [38]. Presented in study were results of high level of HDL and low LDL + VLDL in all experimental groups that were also observed in rats of control groups in the Aberare et al. [39], Kopeć et al. [33] and Silva et al. [40] studies. The increased concentration of glucose was observed in both groups fed diets without addition of lettuce (C and D). It is known that fibre slows up absorption of glucose in the intestine and improves blood glucose control [41]; therefore, the content of glucose was significantly lower in the blood of rats fed biofortified and non-biofortified lettuce. In addition, concentration of triacylglycerols in the serum of these rats was also reduced, compared to the first and the second groups. The activity of ALT and AST enzymes is one of the indicators of liver damage. The diets with addition of both types of experimental lettuce had no effect on liver functions. Kopeć et al. [33] also showed that the level of ALT and AST was not affected by addition of iodine biofortified or non-biofortified lettuce to diets of rats. The level of MDA in the serum and in the liver of rats was not affected by various dietary treatments. Malondialdehyde is an oxidative stress biomarker, and its concentration in an organism increases with increasing degree of lipid peroxidation [42]. Addition of lettuce both enriched with iodine and control to the rats’ diets (D + BL and D + CL) did not generate the oxidative stress in their body.

The second method diagnosis of ID used in this study was estimation of the iodine concentration in urine. The estimation of the iodine nutrition in a human is based on its excretion with urine [2, 4], whereas the concentration of iodine in faeces is not important for evaluation of iodine nutrition. However, the iodine nutrition of rats can be evaluated on the basis of the content of iodine in urine, faeces and organs [43, 44]. Kirchgessner et al. [43] observed that the concentration of iodine in urine, faeces and organs of rats increased with the higher content of iodine in the diet. Therefore, it could be suggested that accumulation of iodine in organs is probably aimed at protection of animals before potential occurrence of ID in food.

When iodine in the diet occurs in the form of I^−^, it is quickly and effectively absorbed in the digestive tract [45]. The iodine in tissues of biofortified vegetables occurs in organic and inorganic forms, but they have not been identified yet. However, it is commonly known that after foliar application of KI on the surface of leaves, I^−^ is absorbed by leaves [46]. The I^−^ uptake by leaves remains mainly in their cells [46, 47], primarily attributable to the cell wall and organelles [14]. Absorption of iodine with these organic structures of plant tissues can be inhibited in the human or animal organism. However, this research has shown that the iodine with biofortified lettuce was more bioavailable than from inorganic KI. The rats fed diet with addition of biofortified lettuce (D + BL) accumulated more iodine in selected organs and excreted less with urine and faeces as compared to the rats fed control diet (C) with addition of KI. Similar tendency was observed in the animal experiment of Kopeć et al. [33].

The excretion of iodine with urine and faeces and accumulation of this trace element in organs were decreased in the D and D + CL groups of rats compared to rats fed control diet and diet with biofortified lettuce (C and D + BL). These results, as well as the increased TSH level in these rats, indicated that the dose of iodine in D and D + CL diets was insufficient for rats. Interesting is the fact that rats fed diet with control lettuce (D + CL) accumulated more iodine in the heart and liver than rats with the D group. It can suggest that absorption of iodine from natural a source (non-biofortified lettuce), besides insufficient iodine dose, was more available than from the D diet.

Tonacchera et al. [48] first showed that iodine from biofortified vegetables is easily bioavailable to the human organism, which indicates that incorporation of biofortified plants into the daily diet can improve human nutrition of iodine. The presented experiment showed evaluation of absorption of iodine in the body of rodents from iodine biofortified lettuce as well as provided an important information about physiological processes in body of rats fed iodine biofortified lettuce.

In summary, spraying KI on lettuce heads in hydroponic cultivation is an effective way to produce lettuce enriched with iodine. Iodine transfer in the food chain from vegetables enriched with this trace element is effective and safe. Iodine with lettuce is bioavailable and easily assimilated by the organism. Biofortified lettuce can be a vehicle of iodine in the daily diet and part of iodine prophylaxis both in developed and developing countries.
